# Assessment of surgical skills on a novel expert-informed immersive virtual reality simulator for distal radius fracture fixation: a cross-sectional validation study

**DOI:** 10.1186/s12909-026-08983-5

**Published:** 2026-03-14

**Authors:** Mads Emil Jacobsen, Monica Ghidinelli, Chitra Subramaniam, Kristoffer Borbjerg Hare, Leizl Joy Nayahangan, Lars Konge, Amandus Gustafsson

**Affiliations:** 1 Center for Orthopaedic Research and Innovation (CORI), Department of Orthopedic Surgery, Naestved Slagelse Ringsted Hospitals, Region Zealand, Slagelse, Denmark; 2 Copenhagen Academy for Medical Education and Simulation (CAMES), Rigshospitalet, Capital Region , Copenhagen, Denmark; 3 Faculty of Health and Medical Sciences, University of Copenhagen, Copenhagen, Denmark; 4 AO Education Institute, AO Foundation, Davos, Switzerland; 5 AO North America, AO Foundation, Wayne, PA USA; 6 Department of Orthopaedic Surgery, Odense University Hospital, Region of Southern Denmark, Odense, Denmark

**Keywords:** Competency-Based Medical Education, Distal radius fracture, Surgical education, Virtual reality simulation

## Abstract

**Background:**

Competency-based medical education requires structured training and valid methods for assessing technical proficiency. Virtual reality simulators offer objective, reproducible assessments but often rely on developer-defined metrics lacking transparency and robust validity evidence. This study aimed to collect validity evidence, guided by Messick’s unified validity framework, for a novel, expert-informed, immersive virtual reality simulator designed to assess proficiency among orthopedic physicians for volar plate fixation of distal radius fractures.

**Methods:**

Twelve orthopedic residents (novices) and eleven experienced orthopedic surgeons completed one distal radius fracture fixation in the simulator. Performance was evaluated using 32 simulator metrics addressing technical proficiency, imaging accuracy, and procedural errors. Simulator metrics were compared between the two groups individually (secondary outcomes) and combined into a composite total simulator score (primary outcome). Validity evidence was structured according to Messick’s framework. The composite score’s discriminatory ability was assessed using the Contrasting Groups’ Method.

**Results:**

Experienced surgeons significantly outperformed novices on nine of 32 simulator metrics, while the novices scored higher on one. After excluding this construct-irrelevant metric, the remaining 31 metrics demonstrated acceptable reliability (Cronbach’s alpha = 0.79). The total simulator score was significantly different for the two groups: novices scored − 1.6 points (CI -12.3 to 9.2), and experienced surgeons scored 61.9 points (CI 53.6 to 70.3), *p* < .001. A discriminatory standard for the total simulator score of 34.0 points completely discriminated between novices and experienced surgeons.

**Conclusion:**

This study provides comprehensive validity evidence – covering all five sources in Messick’s framework – for a newly developed, expert-informed, immersive virtual reality simulator for distal radius fracture fixation. The simulator-based assessment reliably discriminated between novices and experienced orthopedic surgeons. These findings support the simulator’s utility for objective skills assessment in distal radius fracture fixation within orthopedic residency training.

**Supplementary Information:**

The online version contains supplementary material available at 10.1186/s12909-026-08983-5.

## Background

As surgical education shifts to competency-based medical education (CBME), clear learning objectives and robust assessment methods are needed to ensure the development of trainees’ technical proficiency [1]. Simulation-based training (SBT) has become a cornerstone in modern surgical education, providing a safe environment for deliberate practice and skill refinement [2]. Fracture surgery SBT has traditionally used cadaveric and synthetic bone models [3]. Cadavers offer realism but are costly and scarce, while plastic models are more accessible but lack fidelity. Importantly, most fracture fixation procedures also rely heavily on intraoperative fluoroscopy. This is difficult to replicate safely and realistically in most conventional simulation environments due to limited access and radiation restrictions. Additionally, reliance on faculty supervision further limits opportunities for repetition and objective feedback. Together, these factors highlight the need for SBT solutions that integrate imaging and provide standardized assessment without requiring constant faculty presence.

Despite the increasing adoption of surgical simulators, many commercially available systems lack transparency in how performance metrics and scoring systems are developed [4, 5]. Often, these metrics are developer-defined rather than derived through structured, evidence-based methodologies [6]. This raises concerns about whether they accurately measure the intended construct of surgical competence, particularly regarding validity evidence related to content. Although several virtual reality simulators for orthopedic procedures exist, there remains a striking paucity of rigorous validation studies in this field, a limitation extending broadly across the orthopedic specialty [7]. Published evaluations rely on outdated frameworks, if any, and provide only limited validity evidence. Bridging this gap requires assessment frameworks grounded in educational theory and expert consensus. This ensures that simulator-based scores reflect meaningful aspects of operative performance.

Distal radius fracture (DRF) fixation offers an ideal test case for developing and evaluating a simulator-based assessment. DRF is one of the most common orthopedic injuries, leading to substantial patient morbidity and economic burden [8]. Consequently, proficiency in DRF surgery is considered a fundamental skill for graduating orthopedic residents [9]. To our knowledge, no commercially available simulator currently exists for volar locking plate (VLP) fixation of DRFs.

To address these challenges, we developed an immersive virtual reality (iVR) simulator for training and assessing technical competence in DRF fixation using a VLP, based on an assessment tool defined by global expert consensus [10]. The simulator provides a realistic surgical experience. It offers objective, automated performance metrics, unlimited practice, and radiation-free training for intraoperative fluoroscopy. Validity evidence was gathered using Messick’s unified framework, the contemporary standard for assessment validation in medical education [11, 12].

The aim of this study was to collect comprehensive validity evidence for the simulator-based test guided by Messick’s contemporary validity framework while also outlining the expert-informed process used to develop the simulator.

## Methods

This cross-sectional study was conducted between November 2023 and May 2024 at two sites: Orthopedic Department, Slagelse Hospital, Denmark, and the Copenhagen Academy for Medical Education and Simulation (CAMES), Rigshospitalet, Denmark.

### Participants

Orthopedic residents in their first year of specialization formed the novice group. The experienced surgeon group comprised practicing orthopedic surgeons specialized in either hand surgery or orthopedic trauma. They had performed a minimum of 75 VLP fixations for DRFs, either as primary surgeon or supervisor. Although participants were initially to be excluded only if they had used the simulator within the past six months, all included participants were naïve to the iVR simulator. Additionally, novices who had performed more than ten supervised volar locking plate osteosyntheses for distal radius fractures in adults were excluded. All participants completed the procedure individually.

### Sample size

A convenience sample of at least 11 participants per group was used, as no prior data was available on which to base a formal sample size calculation. Considerations related to the central limit theorem informed the decision to analyze group means, and results were interpreted with appropriate caution [13].

### Simulation equipment

**Fig. 1:**
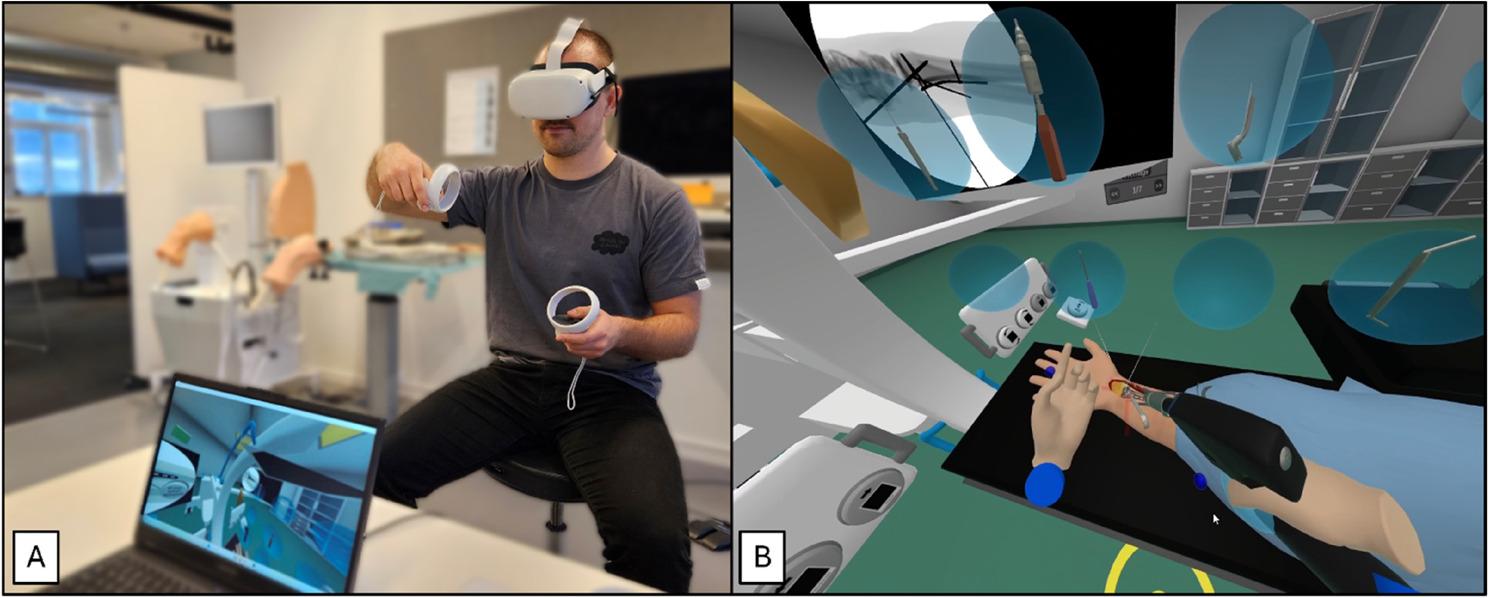
**A**: Participant using the simulation equipment, and **B**: Illustration of the simulated surgery from the user’s perspective

The iVR simulator (VR-BOSS, VitaSim, Odense, Denmark) consisted of a head-mounted display (Oculus Quest 2, Meta Platforms, Inc., Menlo Park, CA, USA), two handheld controllers (Oculus Touch), and a connected laptop (Lenovo Legion i5, Lenovo Inc, Morrisville, NC, USA) (Figure 1). The simulator is commercially available through the company. Participants were immersed in a virtual operating room with a simulated patient positioned for surgery, with the fracture site already exposed. A fluoroscope, and all other equipment necessary for performing the surgery were available in the simulation. The patient’s upper limb could be manipulated to perform anatomical movements of the elbow and wrist joints. The development of the iVR simulator is outlined in Additional file 1, and the simulation is demonstrated in a supplemental video (Additional file 2).

### Simulator assessment

The simulator metrics were defined through a previously published global Delphi consensus study involving AO Trauma faculty and international experts in orthopedic trauma surgery [10]. Through four iterative rounds, the panel achieved consensus on key performance parameters for volar locking plate fixation of distal radius fractures. This included defining optimal values and borderline error thresholds, which were directly implemented as 32 automated simulator metrics. These metrics ensured that performance assessment reflected clinically meaningful and expert-endorsed criteria. These optimal values and borderline error thresholds have not been reported before, and a detailed description of metric derivation and scoring intervals are provided in additional file 1.

### Procedure

Before testing, all participants received an individual, standardized introduction to the simulator followed by a guided warm-up session. Both were guided using prewritten checklists developed specifically for this study to ensure familiarity with simulator functionalities. There was no time-cap, rather all participants had to go through the entire checklist, and time to complete the warm-up was recorded for all participants. Subsequently, each participant performed a single distal radius fracture fixation procedure in the simulator. Participants were required to complete all technical steps of the osteosynthesis. These included fracture reduction, preliminary pinning, and plate fixation (including selection of appropriate plate size, screw types, and screw sizes). Fluoroscopic guidance was used as needed throughout the procedure, followed by a final post-fixation fluoroscopic evaluation. The principal investigator, a physician with experience in VLP surgery, oversaw all procedures, and provided standardized technical assistance limited to verbal clarification and troubleshooting of simulator functions, without any performance-related feedback.

### Variables and outcome measures

Performance was automatically scored by the simulator on 32 predefined simulator metrics covering technical proficiency, imaging accuracy, and procedural errors, as defined by expert input and outlined in Additional file 1 [10]. In addition, the simulator automatically recorded the total time to complete the procedure and number of fluoroscopic images taken. Metrics were scored either as binary (0 or 100 points) or as continuous/interval scales (maximum 100 points, with possible negative scores for severe errors). Individual simulator metric definitions and scoring intervals are detailed in Additional file 1. A composite, total simulator score was calculated as the mean of all included individual simulator metrics, with all metrics contributing equally. This resulted in a theoretical maximum total simulator score of 100 points. Metrics were scored irrespective of missing or unattempted procedural steps. Such omissions affected related downstream metrics and were therefore inherently penalized in the total score. Validity evidence was explored using the framework proposed by Messick [11], which includes five sources of evidence: (1) Content evidence was supported by the expert-informed Delphi process used to define simulator metrics; (2) Response process evidence was ensured through standardized participant instructions, a uniform simulation environment, and automated data capture; (3) Internal structure evidence was examined by exploring internal consistency reliability across simulator metrics; (4) Relations to other variables were evaluated by comparing total simulator scores between novices and experienced surgeons, and (5) Consequential evidence was explored by determining a discriminatory pass/fail standard using the Contrasting Groups’ Method (CGM). This is a well-established standard setting method, in which the performance score distributions of novices and experienced surgeons are plotted, and the intersection point between these distributions defines the cut-score [14]. 

### Statistical analysis

The primary outcome of the study was the difference in total simulator score between novice and experienced surgeons, reflecting the simulator’s ability to discriminate between predefined levels of proficiency. Secondary (exploratory) outcomes included group comparisons for individual simulator metrics, total procedure time, and number of fluoroscopic images taken.

Individual simulator metric scores were compared between groups using Fisher’s exact test for binary metrics. Bootstrapped independent-samples t tests were used for continuous and interval metrics due to non-normal distributions. As a sensitivity analysis, group comparisons for continuous/interval simulator metrics were repeated using the nonparametric Mann-Whitney U test to verify the robustness of results. Individual metric comparisons were treated as exploratory analyses; therefore, we report unadjusted p values. After this exploratory analysis, simulator metrics that demonstrated construct irrelevance were removed before calculating the total simulator score. The internal consistency reliability of the remaining metrics was assessed by calculating a standardized Cronbach’s alpha, as not all metrics could assume negative values. The difference between the novice and experienced groups in the total simulator score was assessed using an independent-samples t test. The distributions of the total simulator scores for both groups were plotted using the CGM [14], and the discriminatory ability of the intersection of these distributions was evaluated. Group differences were considered significant at *p* < .05, and 95% confidence intervals (CI) were reported. Statistical analyses were performed using IBM SPSS Statistics, version 29 (IBM Corp., Armonk, NY, USA).

## Results

**Fig. 2:**
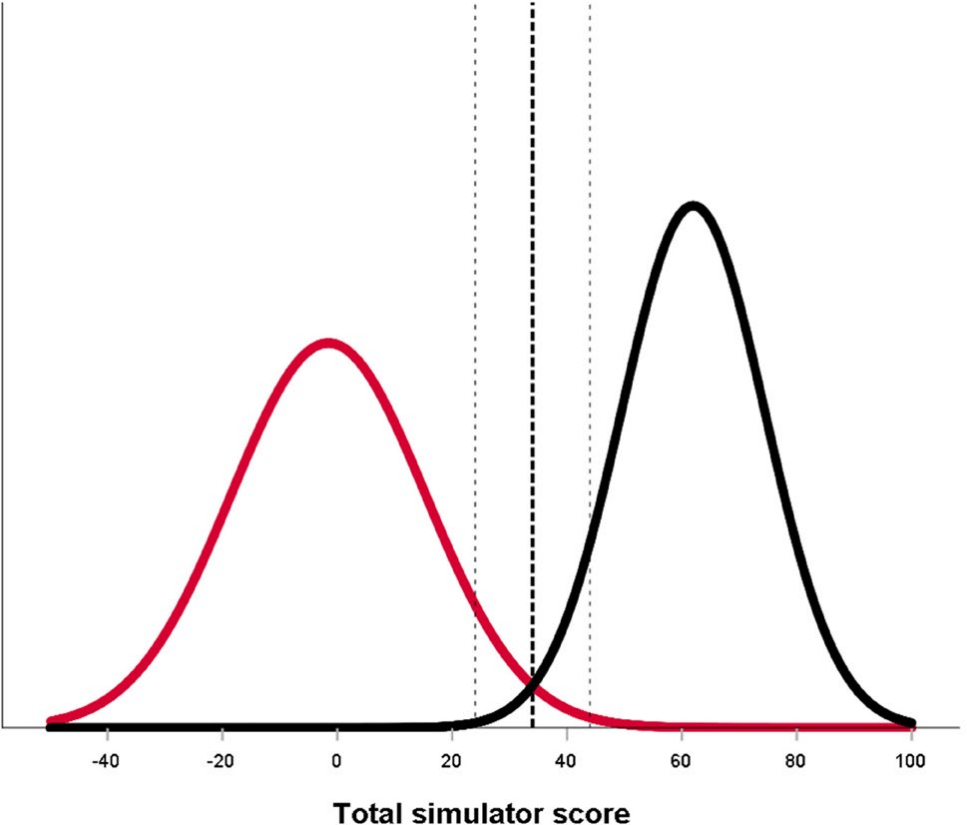
Distribution of total simulator scores for novices (red) and experienced surgeons (black). The bold dotted vertical line represents the intersection of the curves determining the discriminatory standard using the Contrasting Groups’ Method (CGM). The thin dotted vertical lines represent the lower and upper borders of the 95% CI of the intersection

**Fig. 3:**
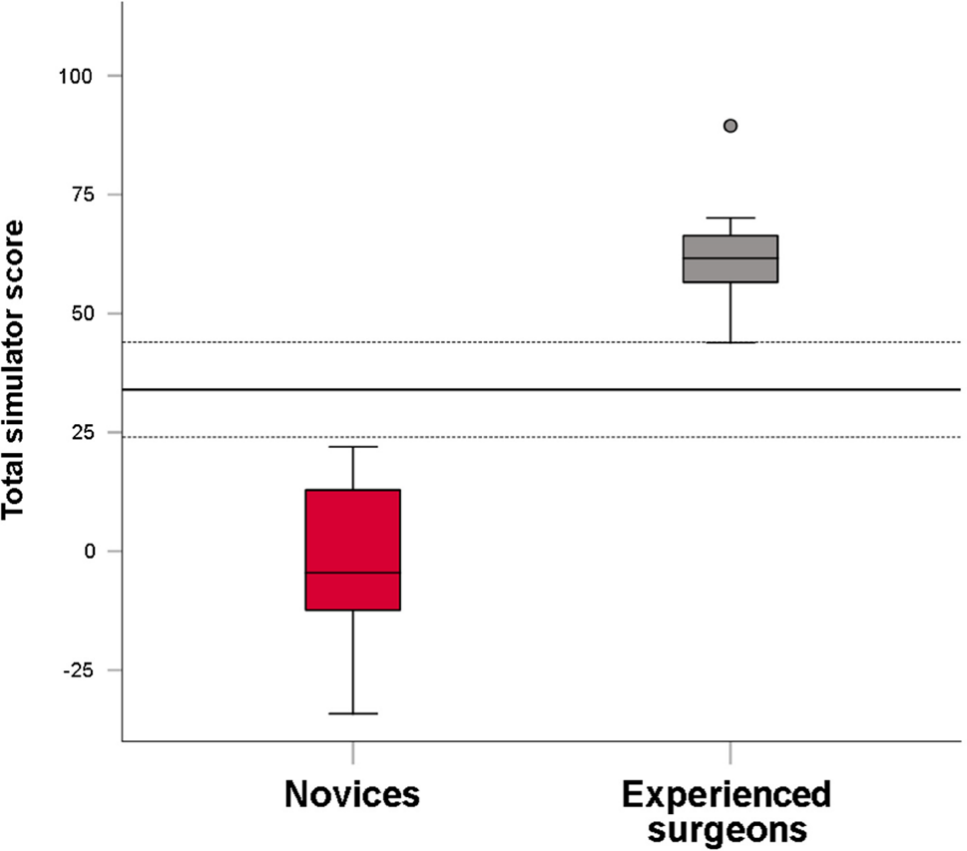
Box plot illustrating total simulator scores for novices (red) and experienced surgeons (grey). The bold horizontal line represents the discriminatory standard as defined by the Contrasting Groups’ Method (CGM). The thin dotted horizontal lines represent the 95% CI for the discriminatory standard. All novices scored below the discriminatory standard and all experienced surgeons scored above the discriminatory standard

Twelve novices from four orthopedic departments and eleven experienced surgeons from seven orthopedic departments participated in the study. Participants’ demographics are summarized in Table 1. Warm-up times were similar between groups: novices, 21 min (CI 18 to 24), and experienced surgeons, 21 min (CI 19 to 23), *p* = .88. The iVR simulator automatically assessed 32 expert-defined performance metrics, as well as time to complete the procedure and number of fluoroscopic images. Tables 2 and 3 compare simulator metric scores between novices and experienced surgeons. Effect sizes (Cohen’s *d* for continuous metrics and φ for binary metrics) ranged from 0.09 to 2.7, consistent with the direction and magnitude of group differences. Experienced surgeons significantly outperformed novices on nine of 32 metrics. Conversely, novices outperformed experienced surgeons on one metric: metric 24 (distance from screw to fracture lines). Because this “reverse-direction” finding indicated construct irrelevance, metric 24 was excluded before calculating the total simulator score, as detailed in the *Discussion* section. There was no significant difference between the groups in time to complete the procedure or number of fluoroscopic images. Nonparametric sensitivity analyses using the Mann–Whitney U test for the continuous/interval simulator metrics confirmed the direction and significance of all main group differences, with only minor variations in p-values. Internal consistency reliability for the remaining 31 metrics was acceptable (standardized Cronbach’s alpha = 0.79). The total simulator scores were normally distributed for both groups. Novices had a mean total simulator score of -1.6 points (CI -12.3 to 9.2), while experienced surgeons scored 61.9 points (CI 53.6 to 70.3), *p* < .001, Cohen’s *d* = 4.2 (CI 2.7 to 5.7). Using the CGM, a discriminatory standard for the total simulator score of 34.0 points (CI 23.9 to 44.1) was identified (Fig. 2), resulting in all novices scoring below, and all experienced surgeons scoring above this threshold (Fig. 3). Validity evidence for the simulator-based test, relating to all five sources within Messick’s validity framework, is summarized in Table 4.

**Table 1:** Participants’ demographic characteristics

Factor	Novices, *n* = 12	Experienced surgeons, *n* = 11
Age, median (IQR)	29 (28–34)	40 (36–42)
Sex, female: male, n	6:6	3:8
Dominant hand, right: left, n	11:1	11:0
Months as orthopedic intern/resident, median (IQR)	10 (8–18)	-
Years as an orthopedic surgeon, median (IQR)	-	3 (1–5)
Experienced surgeons’ subspeciality, orthopedic trauma surgery: hand surgery, n		9:2
Volar plating procedures performed under supervision, median (IQR)	1 (0–6)	-
- Within the last year	1 (0–3)	-
DRF procedures, performed under supervision, by other method than volar plating, median (IQR)	1 (0–5)	-
- Within last year	0 (0–5)	-
Volar plating procedures performed as primary surgeon or supervisor, median (IQR)	-	100 (100–250)
- Within last year	-	15 (10–20)

*IQR * Interquartile range

**Table 2:** Performance scores for binary ordinal simulator metrics

Number	Simulator metric	Novices, *n*=12 correct score (100), *n* (%)	Experienced surgeons, *n*=11 correct score (100), *n* (%)	*P*-value	Effect size, φ
1	Fracture site preparation	10 (83%)	11 (100%)	0.48	0.3
2	Anatomic reduction	3 (25%)	11 (100%)	< 0.001*	0.77
3	Anatomic reduction before plate placement	3 (25%)	11 (100%)	< 0.001*	0.77
4	K-wires intersect with all fragments	4 (33%)	11 (100%)	0.001*	0.70
5	K-wires have not penetrated joint surfaces	3 (25%)	4 (36%)	0.67	0.12
14	Provisional plate fixation	8 (67%)	9 (82%)	0.64	0.17

* *p* < .05

**Table 3:** Performance scores for continuous and interval simulator metrics

Number	Simulator metric	Novices, *n* = 12 Mean score (CI)	Experienced surgeons, *n* = 11 Mean score (CI)	*P*-value	Effect size, Cohen’s d
6	Plate length	87.5 (75.0, 100)	100 (100, 100)	0.08	0.76
7	Plate width	85.4 (75.0, 94.0)	84.1 (77.5, 91.7)	0.83	− 0.09
8	Screw choice	0.0 (-66.7, 63.6)	63.6 (9.1, 100)	0.16	0.61
9	Plate distance to Radiocarpal joint	82.8 (66.1, 94.2)	97.9 (93.1, 100)	0.07	0.79
10	Distance from ulnar border of radius to plate	97.0 (92.9, 99.9)	98.6 (95.2, 100)	0.50	0.29
11	Distance from radial border of radius to plate	45.7 (30.6, 60.7)	53.0 (36.9, 70.1)	0.54	0.26
12	Rotation of the plate	89 (82.5, 95.0)	97.5 (94.8, 99.7)	0.03*	1.0
13	Amount of plate hovering	42.5 (26.6, 55.2)	86.7 (79.9, 91.4)	< 0.001*	2.3
15	Sequence of screw insertion	-52.1 (-91.1, -5 0.0)	43.2 (5.4, 72.5)	0.003*	1.4
16	Holes drilled through dorsal cortex	-408.3 (-522.2, -269.2)	-36.4 (-133.3, 50)	< 0.001*	1.8
17	Holes drilled through radiocarpal joint	58.3 (15.4, 93.3)	100 (100, 100)	0.05	1.8
18	Holes drilled through distal radioulnar joint	100 (100, 100)	100 (100, 100)	1	0.86
19	Choice of drill guide for each hole	41.7 (-33.3, 100)	100 (100, 100)	0.11	0.69
20	Number of screws in each fragment	100 (100, 100)	90.1 (70, 100)	0.34	− 0.44
21	Distance from the distal screws to radiocarpal joint	-167.6 (-240.8, -112.1)	-128.9 (-191.7, -112.1)	0.43	0.34
22	Distance from the distal screws to the distal radioulnar joint	50.0 (25.4, 72.4)	43.0 (22.2, 62.9)	0.69	− 0.17
23	Distance from the distal screw tips to the dorsal cortex	-525.6 (-812.3, -309.1)	-3.3 (-52.3, 45.5)	0.003*	1.5
24	Distance from screw to fracture lines	-403.4 (-606.5, -242.0)	-681.2 (-716.1, -635.5)	0.02*	-1.1
25	Number of proximal screws	89.6 (78.1, 100)	100 (100, 100)	0.10	0.73
26	Distance from the proximal screw tips to the dorsal cortex	62.3 (27.8–85.4)	93.1 (82.4, 100)	0.07	0.81
27	Locking screw tightening	-41.7 (-216.6, 90.9)	81.8 (55.6, 100)	0.17	0.59
28	PA-image	70.8 (52.0, 87.7)	63.3 (19.1, 91.2)	0.74	− 0.14
29	Lateral image	34.8 (-12.7, 76.0)	48.3 (26.4, 70,7)	0.60	0.22
30	Facet image	-19.7 (-44.9, 4.1)	74.4 (64.5, 83.5)	< 0.001*	2.7
31	AP image	-74.1 (-136.3, -19.2)	-41.0 (-114.4, 14.0)	0.49	0.3
32	Tangential image	-155.2 (369.8, 14.0)	-7.8 (-142.6, 63.2)	0.20	0.54
-	Time to complete procedure, minutes	35.5 (29.2, 42.7)	28.6 (25.7, 31.7)	0.1	− 0.71
-	Number of fluoroscopic images	64.3 (43.1, 91.2)	86.4 (68.4, 111.3)	0.19	0.57

* *p* < .05

**Table 4:** Sources of validity evidence according to messick’s framework

Source of evidence	Question related to source of evidence	Validity evidence
Content	Does the content of the test reflect the construct it is intended to measure?	Simulator metrics and scoring intervals were defined by a global expert consensus
Response process	What has been done to reduce bias?	All participants received standardized instructions and were tested in the same setting. The simulator generated objective metric scores.
Internal structure	Is the test score reliable?	Acceptable internal consistency reliability measured by standardized Cronbach’s alpha.
Relationship with other variables	Is there a correlation between test score and a recognized measure of competency?	Experienced surgeons statistically significantly outperformed novices on the total simulator score.
Consequences	What are the consequences of the discriminatory standard?	All novices scored below the discriminatory standard, while all experienced surgeons scored above it.

## Discussion

The aim of this study was to gather validity evidence for the simulator-based test of technical competence in distal radius fracture fixation, guided by Messick’s contemporary validity framework. The study gathered validity evidence broadly across all five sources of Messick’s framework, although the extent of evidence differed between them (Table 4). Though participants completed only a single procedure, the total simulator score reliably discriminated between novices (the intended target group of simulator training) and experienced surgeons, demonstrating a very large effect size. Notably, the discriminatory standard for the total simulator score, derived using the CGM, resulted in no false positives (novices scoring above the standard) or false negatives (experienced surgeons scoring below the standard), which is a noteworthy outcome [10]. Previous orthopedic simulator studies have reported internal consistency coefficients around 0.85–0.88 and effect sizes near 1.9 [15–19]. While lower, our internal consistency reliability (α = 0.79) remains acceptable, and the substantially larger effect size (Cohen’s d = 4.2) indicates a strong discriminatory capacity of the composite simulator score. Details of the structured, global expert-consensus development process that aligned the simulator’s design with educational objectives are provided in Additional file 1.

### Integration within CBME

For a successful transition to CBME, medical education curricula should be developed systematically, following an established framework such as the six-step approach proposed by Thomas, Kern, and colleagues [2, 20]. The first three steps of this approach – conducting ‘*general’* and ‘*targeted needs assessments’*, and formulating ‘*goals and objectives’* – have been addressed in previous studies [10, 21]. This study addresses the next step: selecting iVR simulation as the ‘*educational strategy*’. We chose iVR simulation specifically because it provides an optimal environment for deliberate practice, supports directed self-regulated learning [22], and enables automated objective assessment and feedback. Additionally, this work covers elements of the final steps; *implementation*, by piloting the central part of the curriculum, the iVR simulator, and *evaluation and feedback*, by exploring validity evidence for the simulator-based assessment.

### Simulator development and training implications

Given the central role of assessment in CBME, developing simulators around explicit, evidence-based assessment tools is essential [23]. This approach provides a clear, predefined purpose and aligns software developers, clinical experts, and educational scientists toward a common goal. While details of the simulator development are provided in the appendix, the present report is, to our knowledge, the first to offer comprehensive validity evidence for an orthopedic iVR simulator built through such a transparent process. Our findings support integrating the simulator into early orthopedic training as a structured platform for deliberate practice and assessment, with the potential for performance data to guide individualized feedback and progression toward clinical readiness, pending confirmation from longitudinal training studies.

### iVR in orthopedics

Evidence for the educational value of iVR simulation in orthopedics is growing but has largely centered only on skill-transfer from simulation to cadaver or plastic models [24]. While these findings are important, most studies report neither validity evidence for the underlying simulator metrics nor for the external assessment tools used to evaluate performance on the transfer tasks, and the few that do rely on outdated frameworks [12, 24–27]. Because learners progress at different rates [1], proficiency standards must be established and grounded in rigorous validity evidence for simulator metrics [28, 29]. In the spirit of CBME, such standards ensure that trainees achieve predefined skill levels, irrespective of training duration or the number of training iterations.

### Simulator metrics

We excluded one of the original 32 simulator metrics from the total simulator score. Metric 24 (distance from screw to fracture lines), was excluded because novices scored significantly better than experienced surgeons. Review of performance data and captured videos identified two explanations: first, novices often placed the plate more distally, reducing screw-fracture interference; second, the simulator does not allow users to tactilely identify fracture lines after reduction, and placing screws directly into a fracture line does not cause loss of reduction. This combination of fracture anatomy and lack of haptic feedback likely explains why experienced surgeons – who would likely normally detect such interference tactilely – appeared to perform worse on this metric, despite superior overall technical ability.

In addition to the 32 simulator metrics that were part of our Delphi-derived blueprint, the simulator measured both time to complete the procedure and the number of fluoroscopic images taken, as these parameters are often used as efficiency surrogates in orthopedic simulations. In our study, there was a trend that the experienced surgeons performed the procedure faster than the novices, but it did not reach significance (*p* = .1). Although procedural duration *is* an important consideration in surgical training, it is not meaningful in isolation. As Mazzone et al. highlight, *“… a procedure can be performed quickly but unsafely*,* or phases of the procedure can be omitted resulting in faster completion times”* [30]. Similarly, Gilmer argues that “… *the emphasis should probably be placed more on accuracy*,* than on speed and memorization of steps*,* though in so many of these types of studies*,* we neglect the former for the latter*” [31]. One potential solution could be to include a metric that evaluates procedural quality relative to time; however, we believe that procedural quality should take precedence over procedural speed. Experienced surgeons in our study used more fluoroscopy than novices, aligning with the results of Feeley et al. on a femoral nailing simulator [26]. While minimizing radiation exposure is clinically important, it should not compromise quality of patient care. Also, SBT in acquisition and interpretation of intraoperative fluoroscopic images – integral to the presented simulator – could, over time, reduce exposure by enhancing trainees’ efficiency and accuracy [16].

### Limitations

First, each participant completed only a single repetition of the procedure. Similar to learning curves observed among trainees, a substantial familiarization effect has been reported for experts using surgical skill simulators [32, 33]. Although the discriminatory standard for the total simulator score, derived using the CGM, perfectly distinguished between novices and experienced surgeons in this study, the threshold defining true clinical proficiency would likely be higher if based on performances from surgeons who had undergone sufficient simulator familiarization. This issue is addressed in an ongoing study that includes automated simulator feedback between repetitions.

Second, the modest sample size and recruitment from only two Danish training centers could limit the generalizability of our findings to other educational contexts and healthcare systems. Nevertheless, the sample size is comparable to those in similar studies [25, 26].

Third, we did not adjust for multiple comparisons across the secondary outcomes (individual simulator metrics), which may have increased the risk of type I error; still, inferences are based on the pre-specified primary outcome, the total simulator score.

Finally, current immersive virtual reality technology is constrained by the limited integration of realistic haptics with commercially available handheld controllers. In our simulator, haptic feedback is restricted to vibration during drilling through cortical bone, with no additional tactile cues. This limitation must be acknowledged in simulator and curriculum design, as it narrows the range of skills that can be effectively taught. Tasks requiring nuanced tactile feedback—such as surgical exposure, stability testing of the distal radioulnar joint, and complex fracture reduction—were excluded or simplified. Nonetheless, the immersive environment remains well-suited to developing visuospatial skills. This is particularly true for interpreting two-dimensional fluoroscopic images during the three-dimensional hardware placement, which is a core component of our assessment.

## Conclusions

This study provides comprehensive validity evidence – covering all five sources in Messick’s framework – for an iVR simulator–based assessment of distal radius fracture fixation. After a single repetition, the total simulator score showed acceptable reliability and fully discriminated between novices and experienced surgeons, supporting its use as a pass/fail benchmark in competency-based training.

In perspective, our findings suggest that immersive virtual reality simulation could be a valuable tool for developing technical skills in orthopedic training. Future studies should (1) confirm and refine proficiency cut-scores after repeated exposure and automated feedback, (2) explore the transfer of simulator-acquired skills to the operating theatre, and (3) establish evidence-based strategies for integrating iVR simulation with preparatory instruction and faculty-guided learning within a comprehensive curriculum.

## Supplementary Information

Supplementary Material 1.

Supplementary Material 2.

## Data Availability

The data that support the findings of this study are not openly available due to reasons of sensitivity and are available from the corresponding author upon reasonable request.

## Abbreviations

CBME: Competency-based medical education
SBT: Simulation-based training
DRF: Distal radius fracture
VLP: Volar Locking Plate
iVR: Immersive virtual reality
CGM: Contrasting Groups’ Method
