# Supplementary material for: Assessment of surgical skills on a novel expert-informed immersive virtual reality simulator for distal radius fracture fixation: a cross-sectional validation study
Source: BMC Med Educ. 2026 Mar 14;26:664. doi: 10.1186/s12909-026-08983-5 (PMC13107915; doi:10.1186/s12909-026-08983-5)
Supplement: Supplementary file 2 — Supplementary Material 2. [file 12909_2026_8983_MOESM2_ESM.docx]

Appendix

## Delphi consensus process

The simulator assessment was based on a global Delphi consensus study previously described in detail [1]. This iterative process involved four survey rounds with international AO Trauma faculty and orthopedic trauma experts to define objective performance parameters for volar locking plate fixation of distal radius fractures. The third survey round, which defined the simulator metrics incorporated into the immersive virtual reality (iVR) simulator, is reported here for the first time (response rate 42/98 ≈ 43%). A total of 32 simulator metrics were generated (Figure A.1). Panelists were asked to define optimal values (e.g., screw length in mm) and corresponding borderline error thresholds. A borderline error was defined as a value considered marginally acceptable or marginally erroneous. Parameters unsuitable for continuous scoring were implemented as binary metrics or as mandatory procedural steps. An example of the round 3 survey format is shown in Figure A.2.

## Scoring and metric definition

Optimal intervals for each simulator metric were defined to yield a 100-point score, with linear deductions toward 0 or negative values for deviations from these intervals. The resulting scoring system combined binary, interval, and continuous metrics as outlined below.

- Binary metrics (Items 1 – 5 and 14): Correct performance = 100 points; incorrect or unattempted = 0 points.
- Interval metrics (Items 6 – 7 and 25): Expert-defined “optimal” and “borderline” performance corresponded to 100 and 50 points, respectively. Intermediate performances were linearly interpolated (Figure A.3).
- Continuous metrics (Items 9 – 13, 21 – 26): Expert mean ± 1 SD defined the optimal range performance, yielding 100 points. Expert defined “borderline” performance corresponded to 50 points and intermediate performances were linearly interpolated (Figure A.4).
- Sequence metric (Item 15): The correct sequence of screw insertion was defined by expert ranking (Figure A.5). Deviations from this order reduced the score by 25 points per step.
- Fluoroscopy metrics (Items 28 – 32): Derived from a separate validation study [2].
  - For three projections with established validity, optimal intervals were based on the z-score distributions of experienced surgeons from that study.
  - For the two remaining projections, reference values were defined using anatomical literature [3,4], as these views have well-established clinical utility.

## Total simulator score

The composite simulator score was defined as the mean of all individual metric scores that remained after exclusion of construct-irrelevant items. Because metrics were scaled from –∞ to 100 or 0–100, negative totals were possible for cumulative severe penalties. A total score of 100 represented flawless performance across all domains, while lower scores indicated greater deviation from expert-defined standards. This composite score served as the study’s primary outcome measure.

## Simulator development

The immersive VR simulator (Virtual Reality Basic Osteosynthesis Surgery Simulator, VR-BOSS; VitaSim, Odense, Denmark) was developed using the Unity platform (Unity Technologies, San Francisco, CA, USA). Software development followed an iterative, collaborative process involving software engineers, medical education specialists, and orthopedic surgeons (authors MEJ, KBH, AG).

### Modeling of bone, fracture, and hardware:

- The virtual model included the distal humerus, radius, ulna, carpus, and hand, reconstructed from a CT scan of an uninjured right upper limb.
- A simple intra-articular distal radius fracture (AO/OTA 2R3C1.2.t) with a stable distal radioulnar joint was incorporated, matching the case used in the third Delphi round.
- The simulated implant system replicated the DePuy Synthes Variable Angle LCP® Two-Column Volar Distal Radius Plate 2.4 and the DePuy Synthes Small Fragment System instruments.

## References

1. **Jacobsen ME, Nayahangan LJ, Ghidinelli M, et al.** Assessment of Technical Competence in Distal Radius Fracture Fixation by a Volar Locking Plate: A Global Delphi Consensus Study. *J Hand Surg Am*. 2023;48(9):875-885. doi: 10.1016/j.jhsa.2023.05.012.
2. **Sønderup M, Gustafsson A, Konge L, Jacobsen ME.** Intraoperative fluoroscopy skills in distal radius fracture surgery: valid and reliable assessment on a novel immersive virtual reality simulator. *Acta Orthop*. 2024;95:477-484. doi:10.2340/17453674.2024.41345
3. **Medoff RJ.** Essential radiographic evaluation for distal radius fractures. *Hand Clin*. 2005;21:279-288. doi: 10.1016/j.hcl.2005.02.008
4. **Haug LC, Glodny B, Deml C, et al.** A new radiological method to detect dorsally penetrating screws when using volar locking plates in distal radial fractures. The dorsal horizon view. *Bone Joint J*. 2013;95-B:1101-1105. doi: 10.1302/0301-620X.95B8.31301.

**Figure A.1**. Delphi-study assessment parameters and corresponding simulator metrics. Figure adapted from Figure 3 in: Jacobsen et al. Assessment of Technical Competence in Distal Radius Fracture Fixation by a Volar Locking Plate: A Global Delphi Consensus Study. J Hand Surg Am. 2023 Sep;48(9):875-885. Licensed under CC-BY 4.0 https://doi.org/10.1016/j.jhsa.2023.05.012


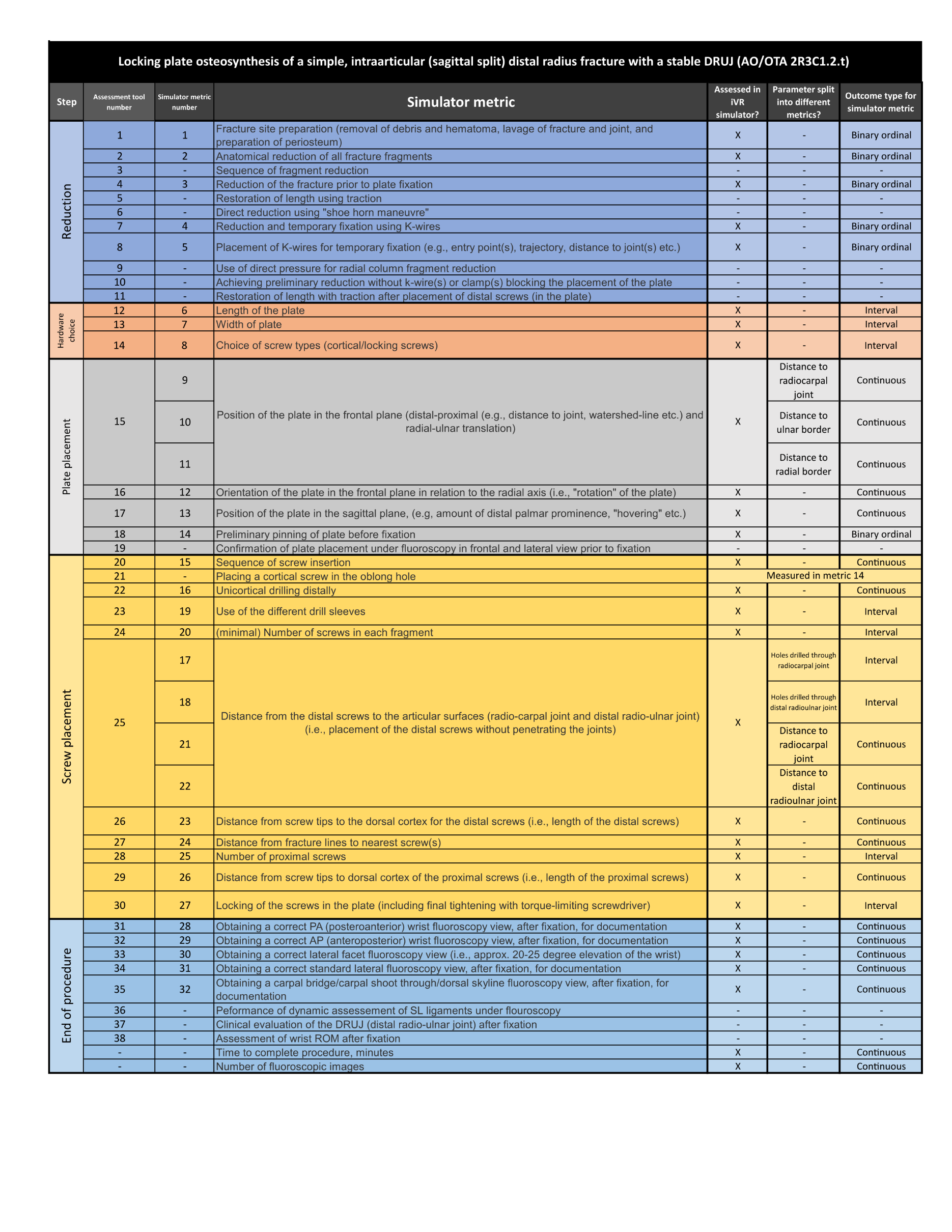


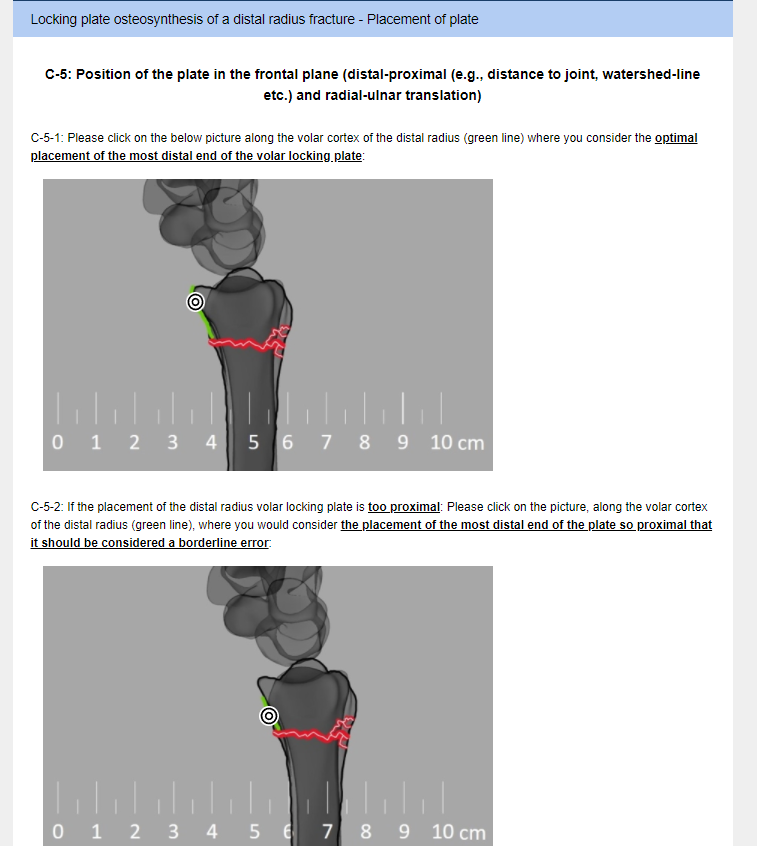
**Figure** **A.2.** Example of round 3 survey. An input from one of the experts in the panel. A) optimal value definition., B) borderline error value definition.


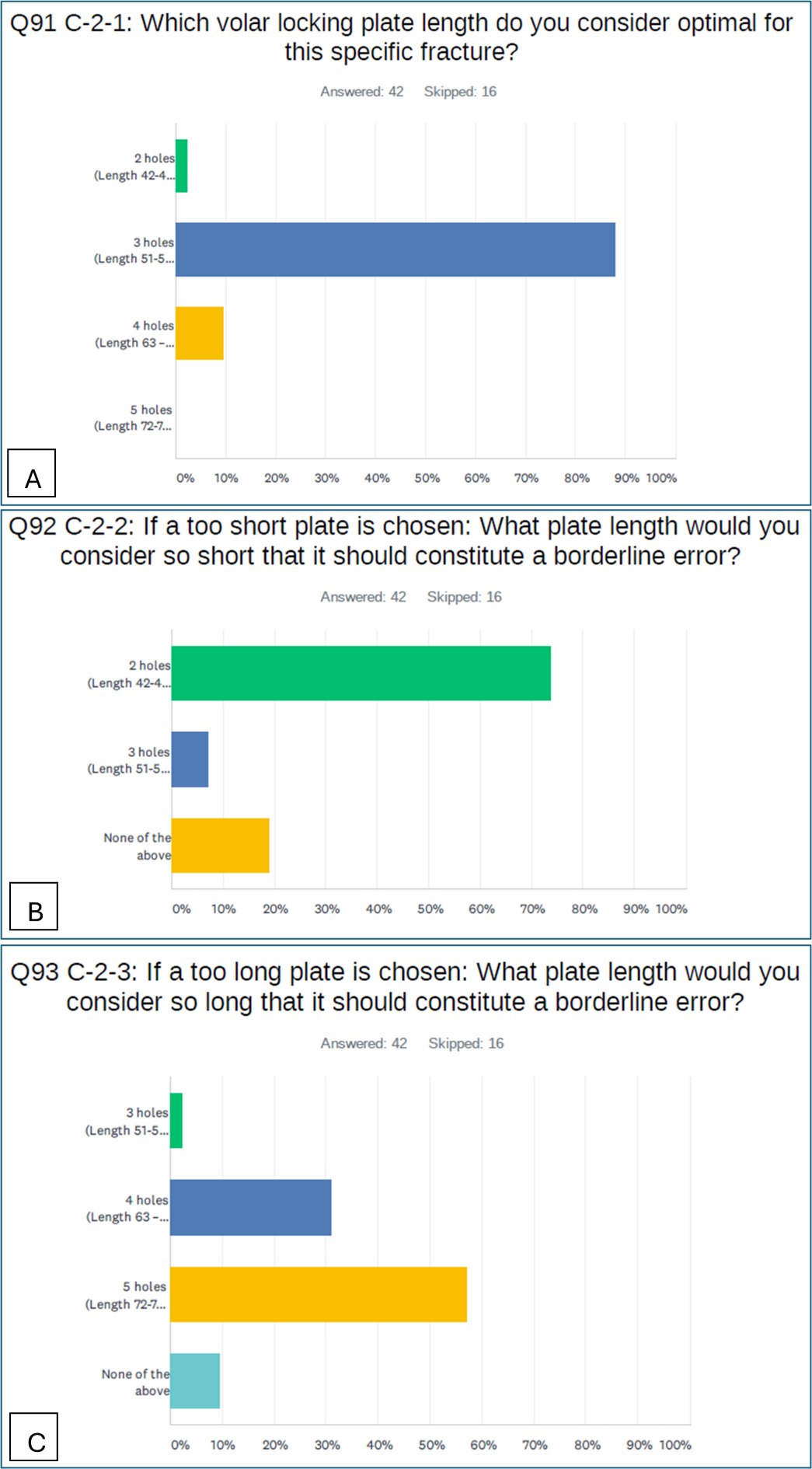
**Figure** **A.3.** A) optimal choice. B) and C) borderline error values.


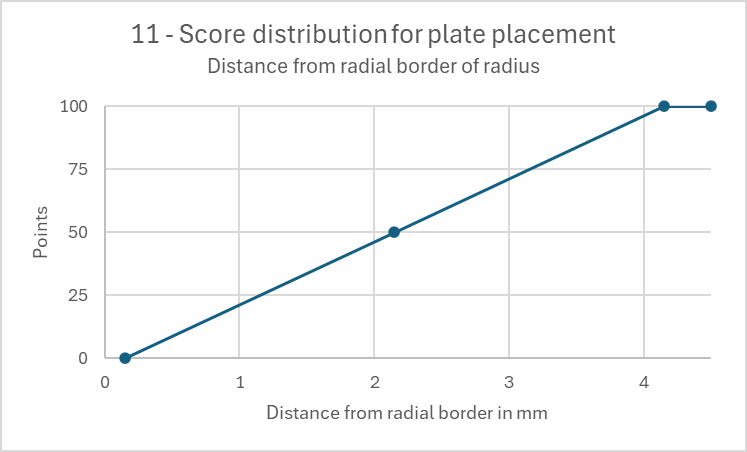

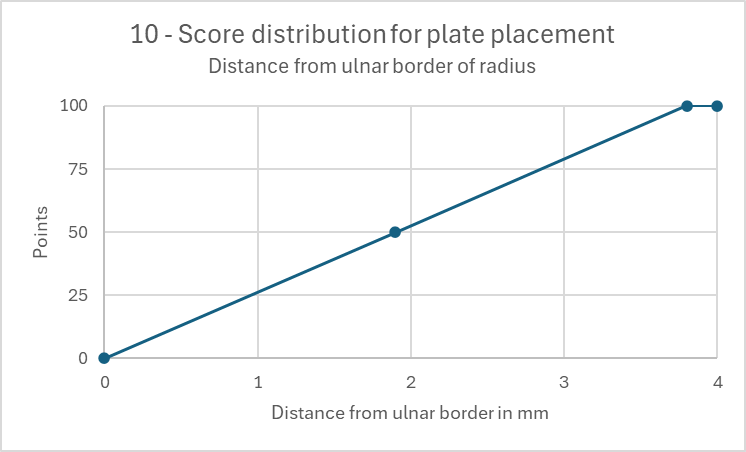

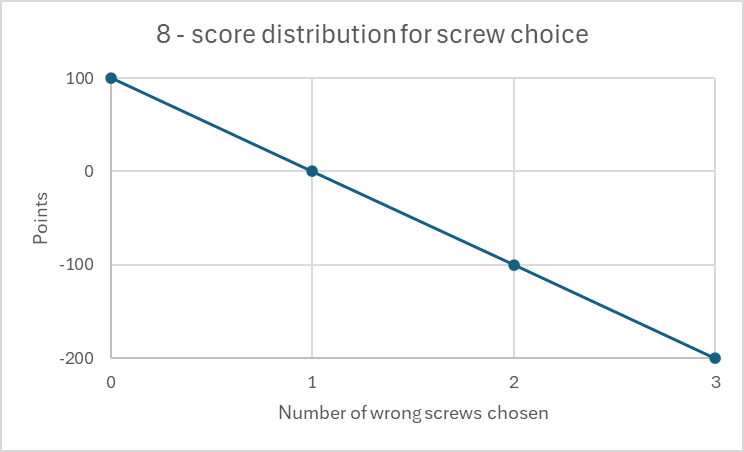

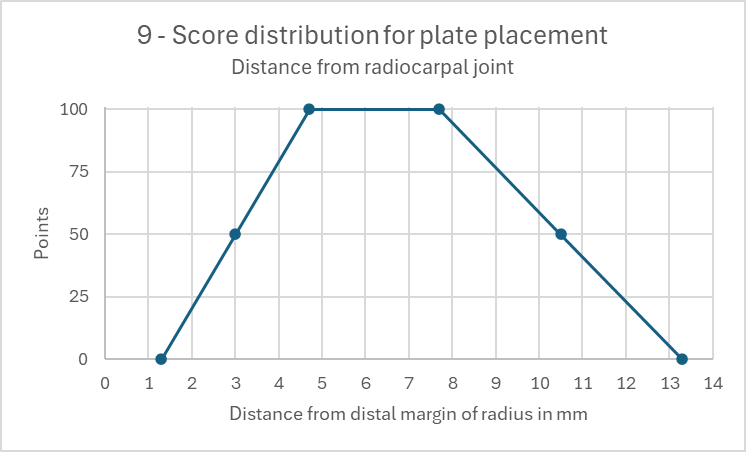

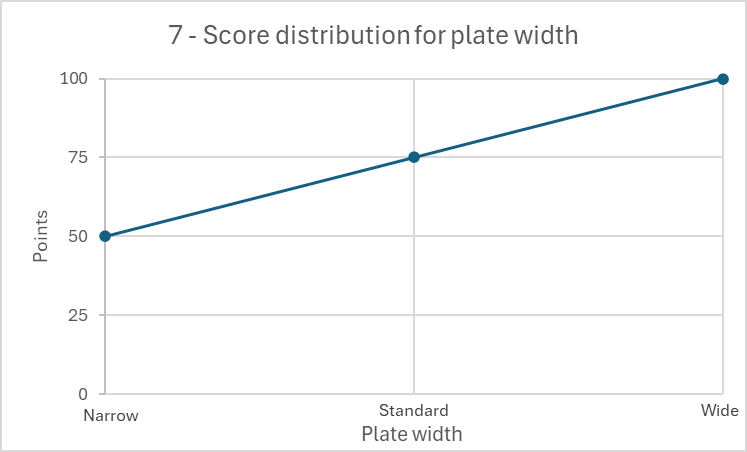

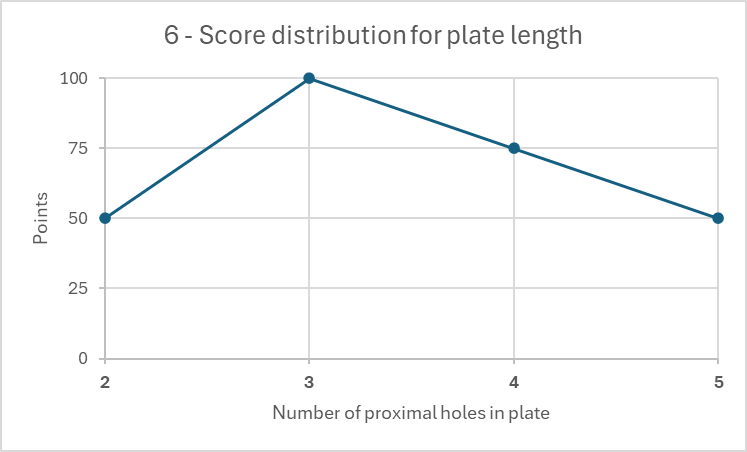


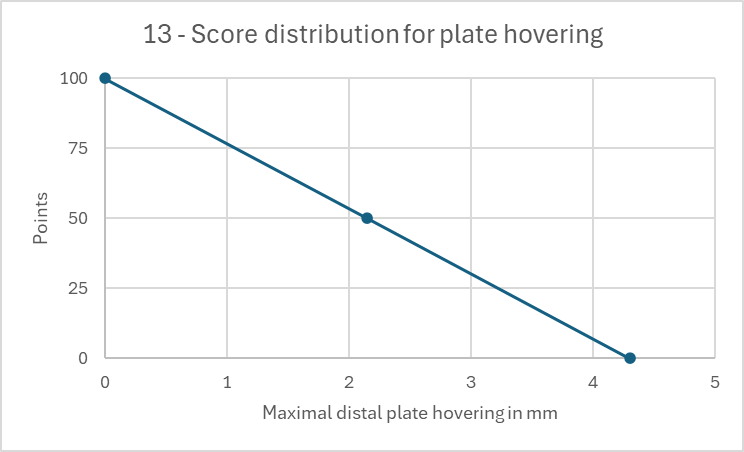

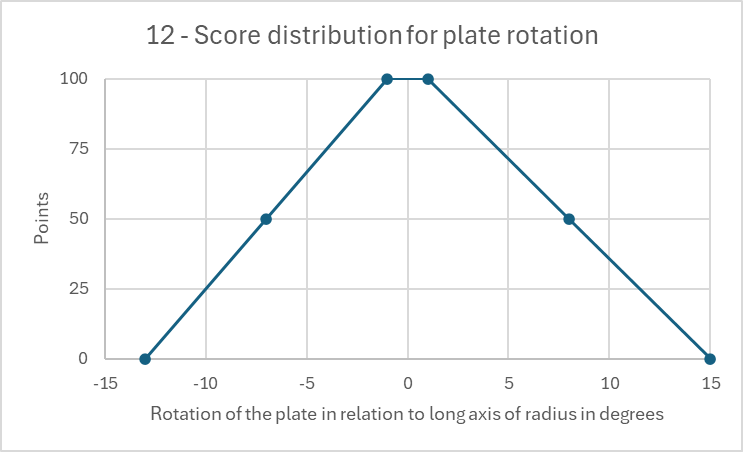


**Figure A.4**. Score distribution curves for interval and continuous simulator metrics.


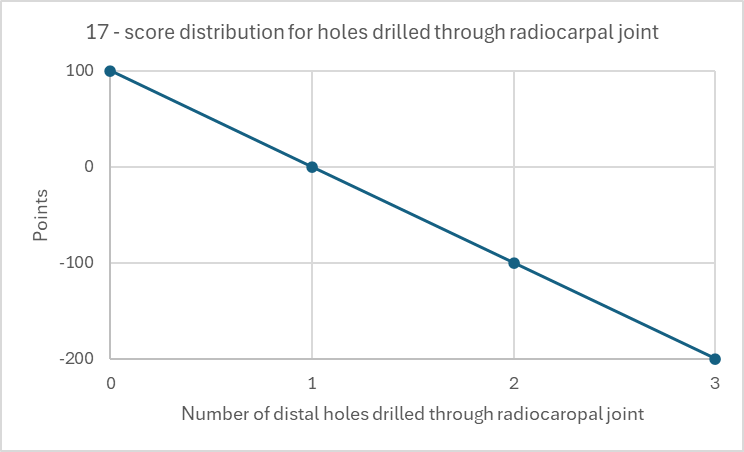

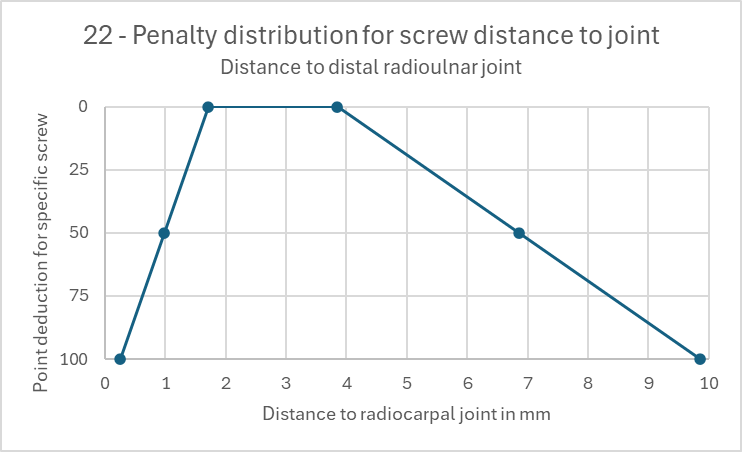

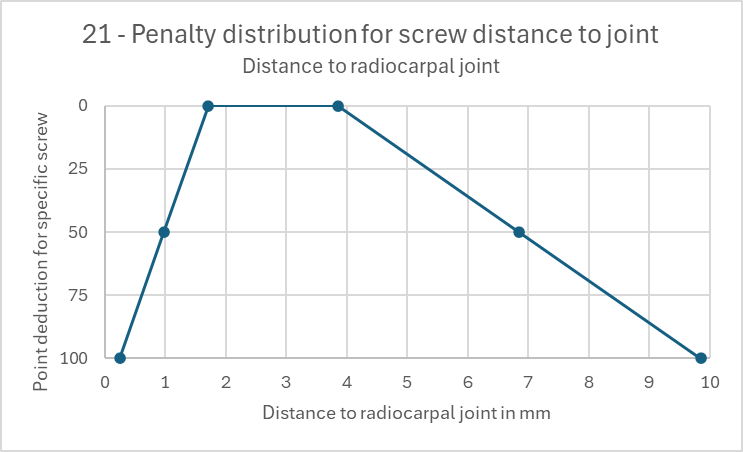

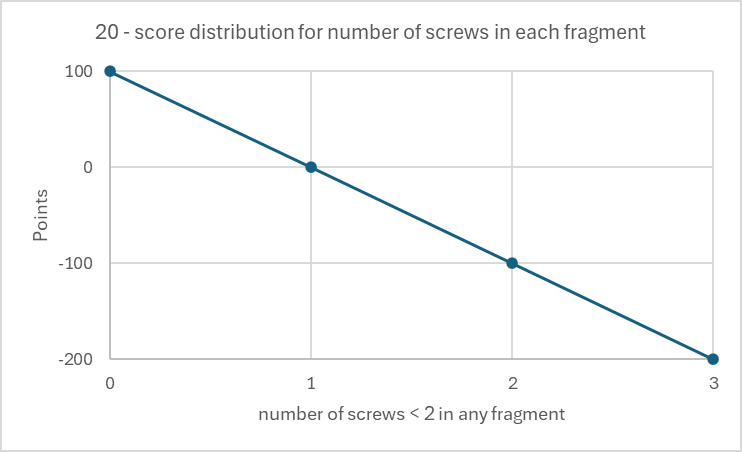

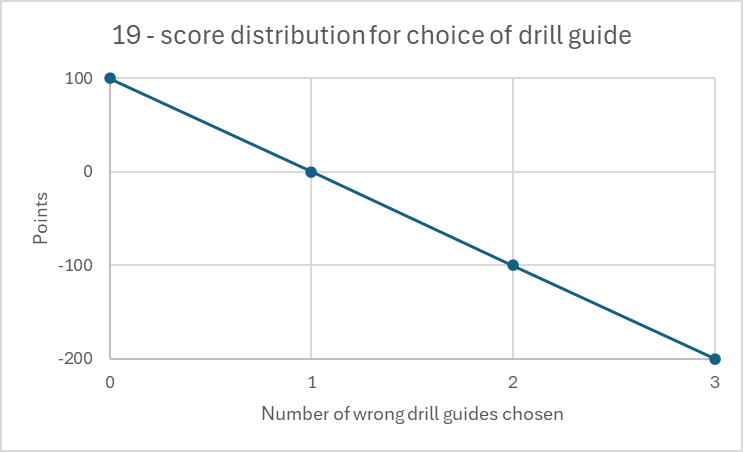

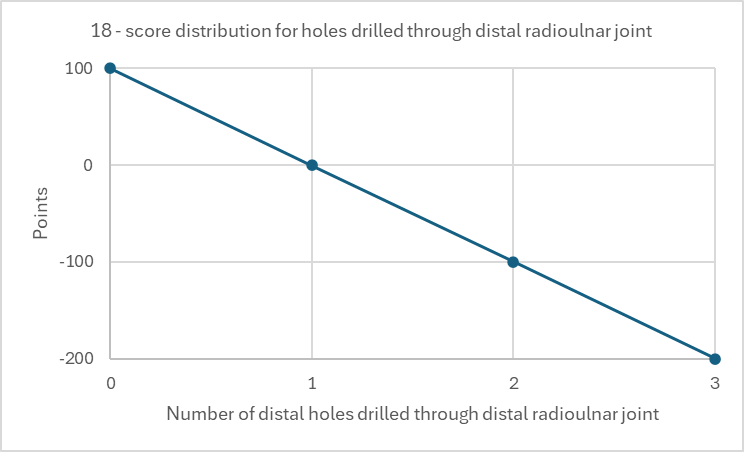

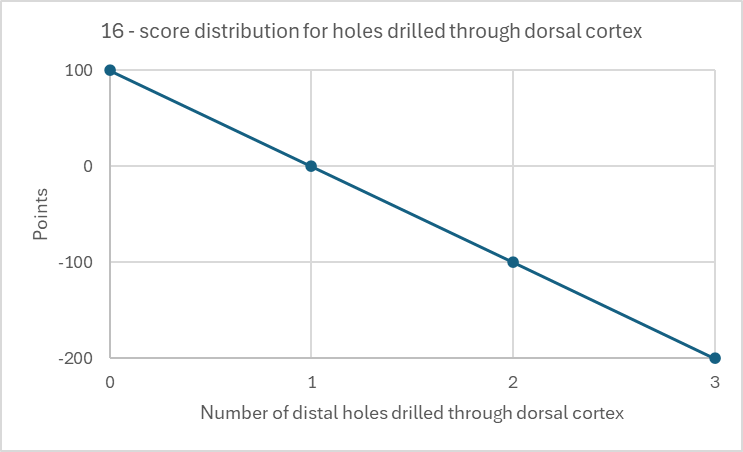

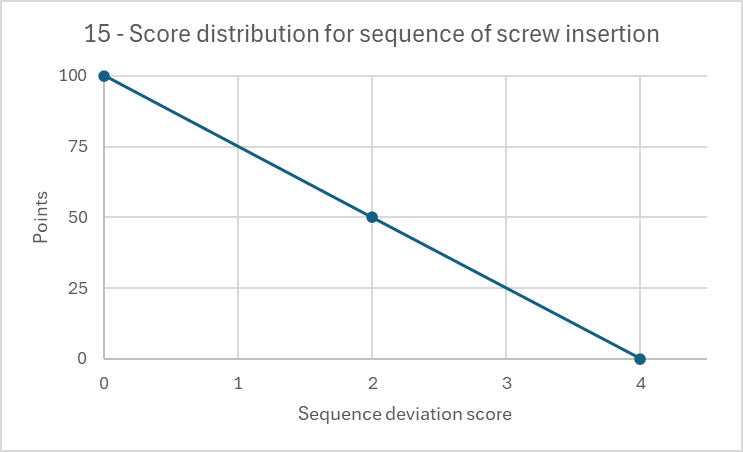


**Figure A.4**. Score distribution curves for interval and continuous simulator metrics continued.


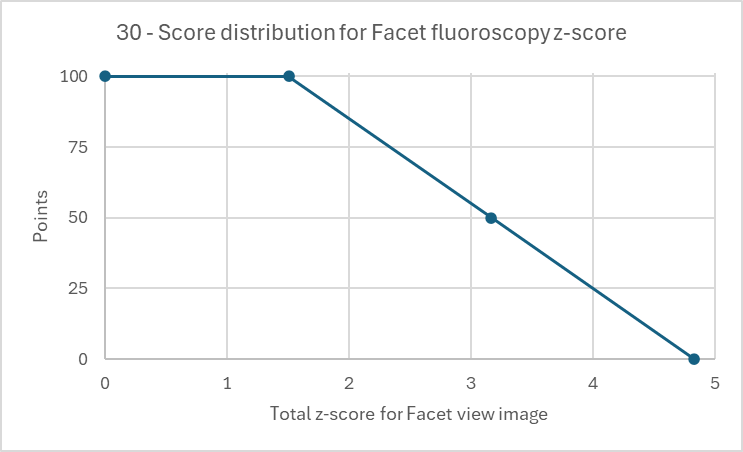

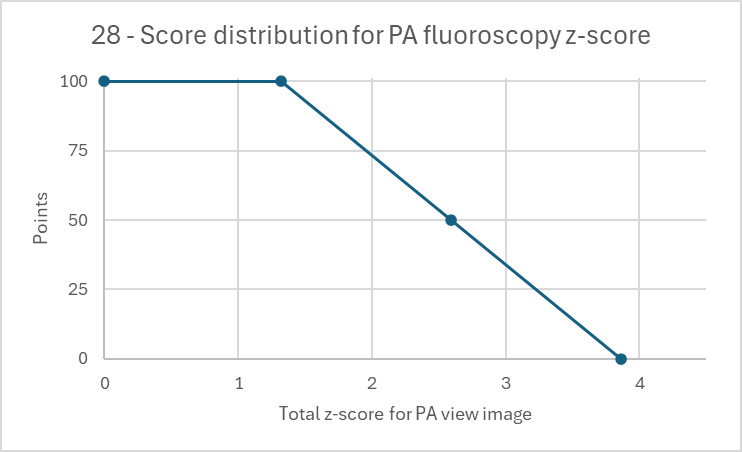

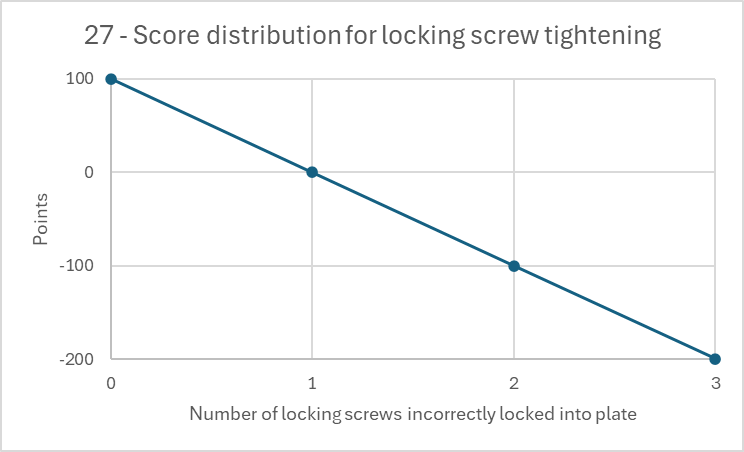

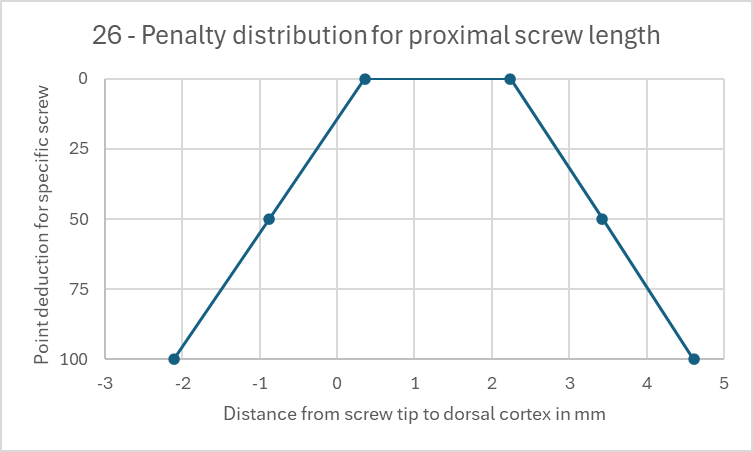

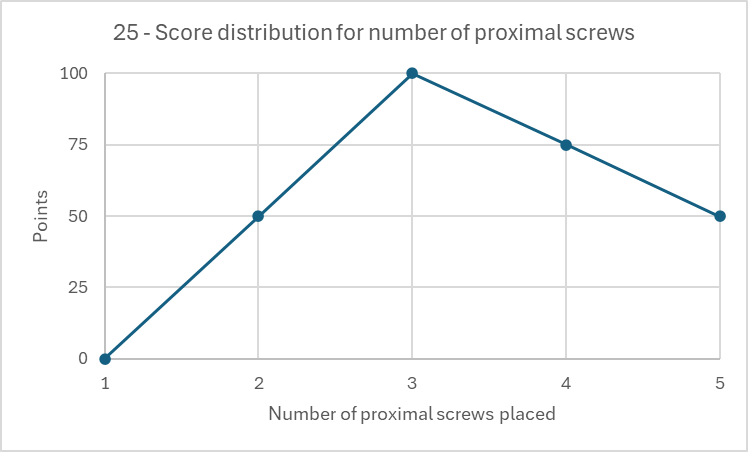

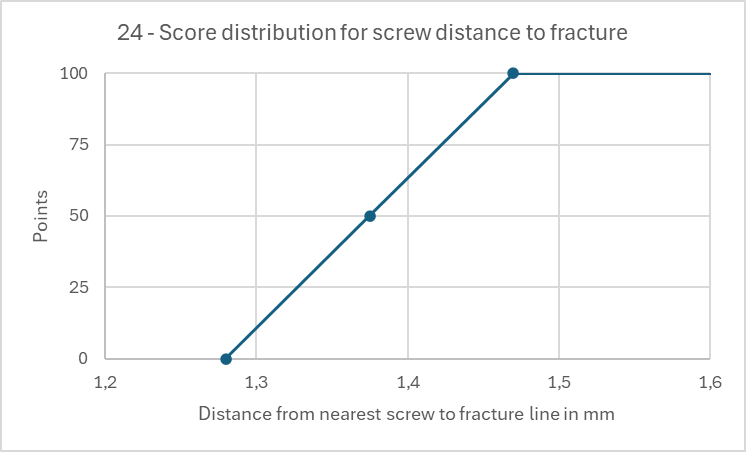

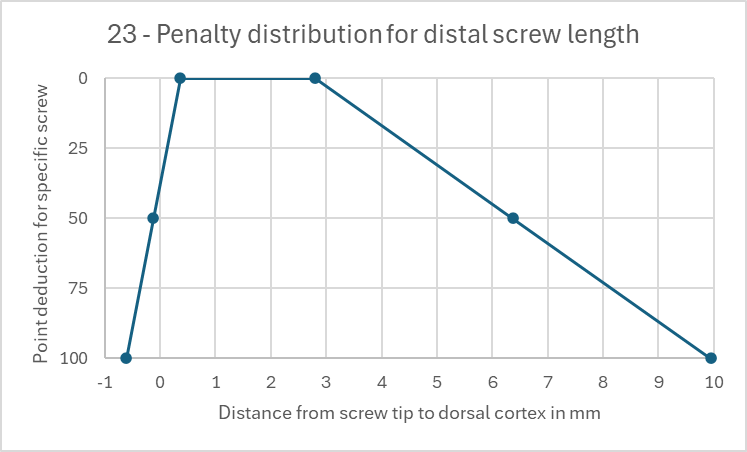


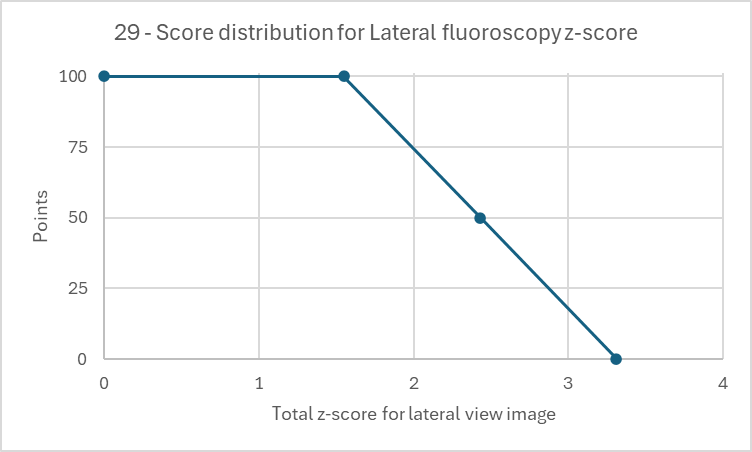


**Figure A.4**. Score distribution curves for interval and continuous simulator metrics continued.


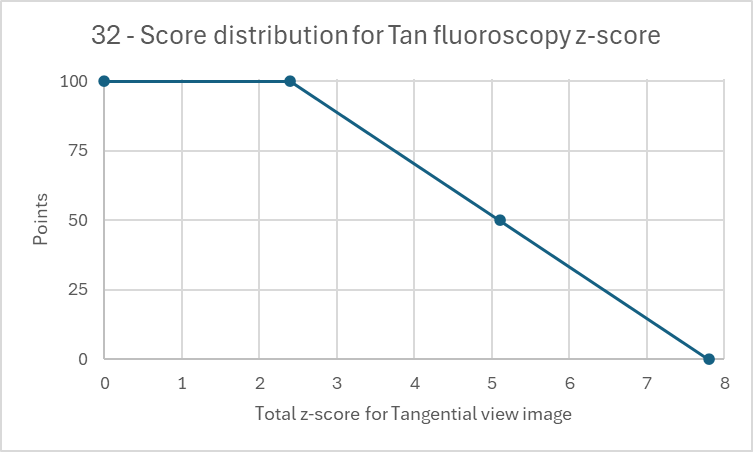

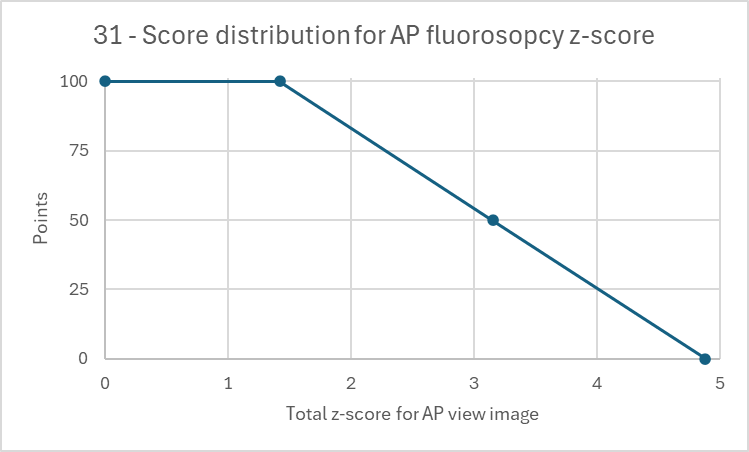


**Figure A.4**. Score distribution curves for interval and continuous simulator metrics continued.
